# Transcriptome Dynamics in the Developing Larynx, Trachea, and Esophagus

**DOI:** 10.3389/fcell.2022.942622

**Published:** 2022-07-22

**Authors:** Kristy D. Wendt, Jared Brown, Vlasta Lungova, Vidisha Mohad, Christina Kendziorski, Susan L. Thibeault

**Affiliations:** ^1^ Department of Surgery, Division of Otolaryngology, Head, and Neck Surgery, University of Wisconsin, Madison, WI, United States; ^2^ Department of Biomedical Engineering, University of Wisconsin-Madison, Madison, WI, United States; ^3^ Department of Statistics, University of Wisconsin-Madison, Madison, WI, United States; ^4^ Department of Biostatistics and Medical Information, University of Wisconsin-Madison, Madison, WI, United States

**Keywords:** RNAseq, development, larynx, morphogenesis, esophagus, trachea

## Abstract

The larynx, trachea, and esophagus share origin and proximity during embryonic development. Clinical and experimental evidence support the existence of neurophysiological, structural, and functional interdependencies before birth. This investigation provides the first comprehensive transcriptional profile of all three organs during embryonic organogenesis, where differential gene expression gradually assembles the identity and complexity of these proximal organs from a shared origin in the anterior foregut. By applying bulk RNA sequencing and gene network analysis of differentially expressed genes (DEGs) within and across developing embryonic mouse larynx, esophagus, and trachea, we identified co-expressed modules of genes enriched for key biological processes. Organ-specific temporal patterns of gene activity corresponding to gene modules within and across shared tissues during embryonic development (E10.5-E18.5) are described, and the laryngeal transcriptome during vocal fold development and maturation from birth to adulthood is characterized in the context of laryngeal organogenesis. The findings of this study provide new insights into interrelated gene sets governing the organogenesis of this tripartite organ system within the aerodigestive tract. They are relevant to multiple families of disorders defined by cardiocraniofacial syndromes.

## Introduction

Mammalian breathing and swallowing are closely interrelated in their central and muscular control, and the esophagus, trachea, and larynx share origin and proximity within the foregut endoderm during embryonic development. The larynx is situated at the crossroads of the upper aerodigestive tract cranial to the developing esophagus and trachea. It participates in both respiratory and alimentary functions throughout development, with roles in respiratory maintenance and swallowing in fetal stages that expand to include protection of the lower airway and phonation abruptly after birth through adulthood (Harding, 1984; Harding, Bocking, and Sigger, 1986; Miller, Sonies and Macedonia, 2003). The larynx, trachea, and esophagus share progenitor field specification signaling mechanisms, though relatively little is known about the underlying genetic characteristics of organogenesis considering the shared origin and interrelation of all three distinct tissues in the upper aerodigestive tract (Billmyre, Hutson and Klingensmith, 2015; Lungova et al., 2015; Lungova and Thibeault, 2020; Teramoto et al., 2020). Identifying genes with correlated expression within each organ through development from a shared origin can shed more light on their possible functions, as genes with similar expression patterns may be functionally related (Zhang and Horvath, 2005; Farhadian et al., 2021).

Here, we characterize transcriptional dynamics during development of the esophagus, trachea, and larynx by conducting a time-series study of *in vivo* developmental gene expression using a statistical analysis pipeline informed by weighted correlation network analysis (WGCNA). High-throughput whole-transcriptome sequencing *via* RNA-seq can help comprehensively describe gene expression profiles in multiple tissues over time. Single-cell RNA-seq technology combined with differential gene expression analysis has been used previously to describe the transcriptional specification of the mammalian trachea and esophagus (Kuwahara et al., 2020), probe expression signatures of the esophageal epithelium (Owen et al., 2018; Busslinger et al., 2021) and unravel cell lineage relationships in the respiratory tract (Treutlein et al., 2014; Plasschaert et al., 2018; Zaragosi, Deprez and Barbry, 2020). These analyses focus on differentially expressed gene screening. However, shared gene sets between organs remain unexplored, as do tissue-specific yet functionally interrelated gene sets within all three tissues through developmental time. Temporal differences in gene expression levels are described across and within the larynx, esophagus, and trachea in terms of the phenotypes of interest, including neuron fate specification, muscle development, chondrification, epithelium development, and motor-driven movement. Esophageal atresia (EA), tracheoesophageal fistula (TEF), congenital laryngeal webbing, and/or laryngeal cleft are congenital anomalies of unknown molecular etiology. They often occur together or alongside associated cardiac abnormalities, including ventricular septal defects and Tetralogy of Fallot, as well as limb abnormalities *via* VACTERL association (Greenwood and Rosenthal, 1976; Walner et al., 1999; Leboulanger and Garabédian, 2011; O’Shea et al., 2020). The findings of this study provide new insights into the interrelated gene sets governing the organogenesis of this tripartite organ system. They further characterize differentially expressed genes (DEGs) enriched for cardiogenesis processes concomitant with the formation of the laryngo-tracheoesophageal septum and realization of the vocal folds.

## Results

### Identification of Differentially Expressed Genes Within and Across the Larynx, Esophagus, and Trachea

**FIGURE 1:**
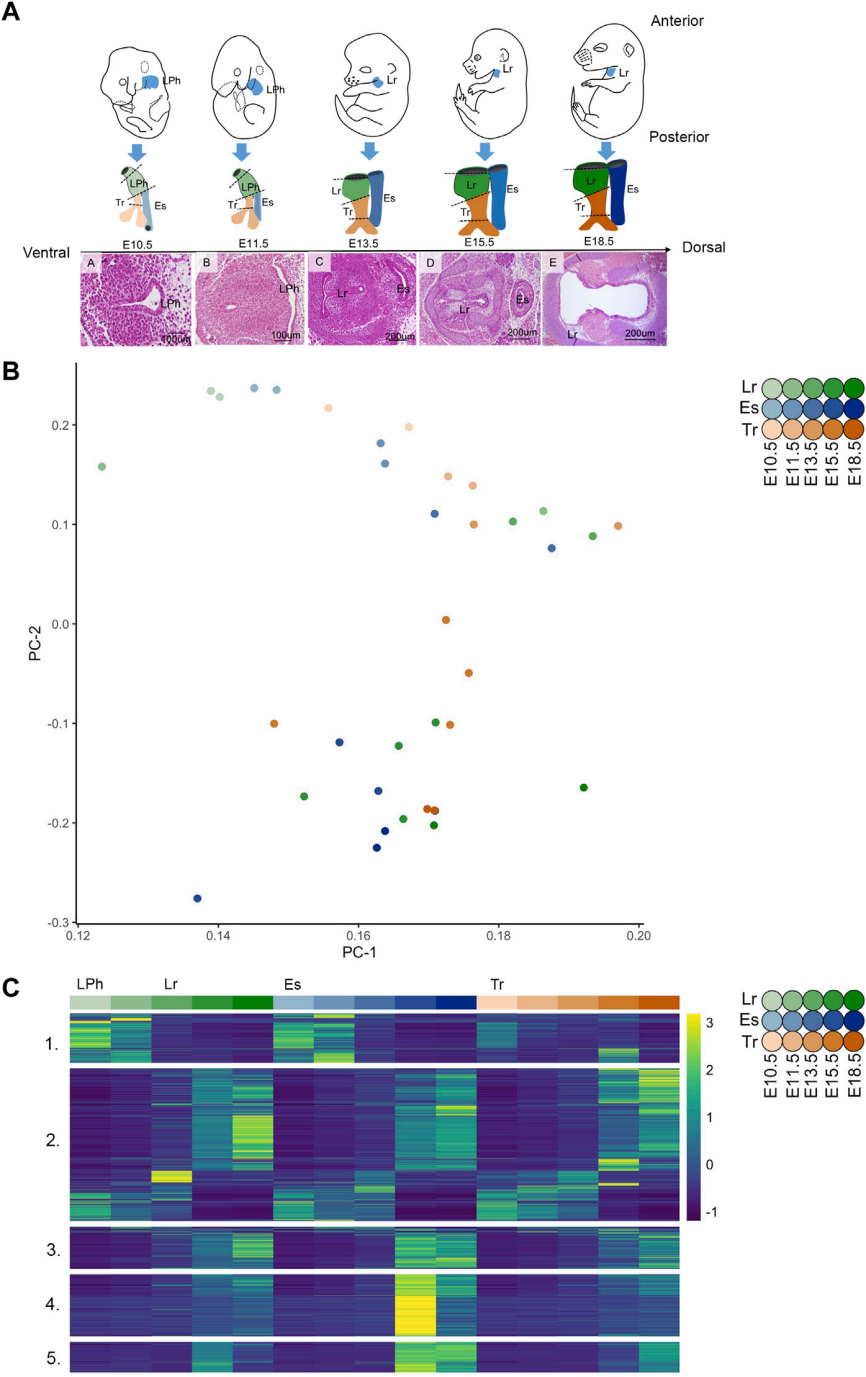
Global transcriptome structure across the esophagus, trachea, and larynx during embryonic development, **(A)** Schematic of developing embryos shows the color key for organ identity and developmental stage across the study timespan above hematoxylin and eosin histological sections at the level of the developing vocal folds during each time point. **(B)** PCA analysis demonstrates 2D representation of whole-tissue transcriptome principal components at E10.5, E11.5, E13.5, E15.5, and E18.5 across embryonic development of the Lr, larynx; Es, esophagus; Tr, trachea; **(C)** Heatmap of differentially expressed genes (DEGs) (*n* = 3,472) constructed from normalized expected counts across embryonic development of the primitive laryngopharynx (LPh), larynx (Lr), esophagus (Es), and trachea (Tr) at E10.5, E11.5, E13.5, E15.5, and E18.5 with expression measures averaged across replicates within each tissue/age combination and then converted within each gene into a z-score for improved visual comparison.

Our main objectives were to 1) identify DEGs to elucidate transcriptome variation within and across the esophagus, trachea and larynx during key timepoints in embryogenesis and 2) characterize the influence of postnatal development and maturation on laryngeal tissue transcriptomics as the organ abruptly initiates protection of the lower airway and phonation at birth (German and Palmer, 2006). For all analyses, 53,647 Ensemble genes were mapped to 18,032 human orthologs in gene symbol format, with details in the quality control and gene expression quantification section of Methods. Defining DEGs as significantly varied gene expression through prenatal timepoints embryonic day (E)10.5, E11.5, E13.5, E15.5, and E18.5, we identified a total of 3,472 (p-adj ≤ 0.01) DEGs shared across all three tissues during embryogenesis. Significantly varied genes within the esophagus and trachea through prenatal timepoints E10.5, E11.5, E13.5, E15.5, and E18.5 were considered, and 2,766 and 2,921 DEGs (p-adj ≤ 0.01) were identified. Analysis of significantly varied genes within the larynx across development incorporated birth (P0) and adult (4–6 weeks) timepoints, such that E10.5, E11.5, E13.5, E15.5, E18.5, P0, and adult timepoints were all considered, with the 4,519 DEGs (p-adj ≤ 0.01) resulting from the within-tissue laryngeal analysis Figure 1.

The developmental timespan from mid-gestation E10.5–E18.5 encompasses much of the development of the upper aerodigestive tract. In mice, between E9.5 and E11.5 (equivalent to weeks four to six human gestation), a compartmentalization process occurs with the formation of the respiratory diverticulum from the ventral anterior foregut endoderm and the gradual separation of the ventral respiratory diverticulum from the dorsal anterior foregut by the tracheoesophageal septum. The primitive LPh is anteriorly continuous with the newly-formed esophagus and trachea at E10.5, shown in Figure 1A, as an anatomical schematic juxtaposed with transverse hematoxylin and eosin histological sections. As the lung bud elongates at E11.5, tracheoesophageal septation, initiated at ∼9.5, proceeds anteriorly toward the primitive LPh (Qi and Beasley, 2000; Billmyre, Hutson, and Klingensmith, 2015) in synchrony with the fusion of the lateral walls of the LPh. The laryngotracheal tube separates from the esophagus *via* laryngotracheo-esophogeal septation at E13.5 at the level of the developing vocal folds in the larynx, such that the posterior part of the larynx is ventrally continuous with the trachea and dorsally continuous with the septum at E13.5 shown in Figure 1A. During vocal fold development, septation of the laryngotracheal tube and esophagus occurs in synchrony with the initiation of epithelial lamina (EL) recanalization, cartilage chondrification, intrinsic muscle formation, and the initiation of a diminution in cell proliferation machinery that continues through E18.5 in developing embryos (Lungova et al., 2015; Griffin et al., 2021).

### Global Transcriptome Structure Across Embryonic Esophagus, Trachea, and Larynx

The highest variance components of gene expression (calculated from the 3,472 DEGs shared across all tissues) across all samples define common, age-dependent changes across all three tissues, shown as a PCA plot in Figure 1B. Tissue-related effects were negligible between groups in these first two principal components. The earliest and latest timepoints occupied opposite ends of the second principal component in the esophagus, trachea, and larynx, with the E13.5–E15.5 timepoints dominating expression changes in the principal component that most reflects gene changes due to age (PC2). Also, after DE testing, the 3,472 DEGs across all tissues were clustered into co-expressed groups using the WGCNA gene network identification pipeline consisting of correlation, Topological Overlap Measure (TOM), and hierarchal clustering (Zhang and Horvath, 2005; Yip and Horvath, 2007; Langfelder and Horvath, 2008; Song, Langfelder and Horvath, 2012). DEGs across the primitive laryngopharynx/larynx, esophagus, and trachea are clustered into the five co-expressed modules and displayed as a heat map of normalized expected counts (ECs) in Figure 1C, with select genes from the top gene ontology (GO) terms of each module shown in Supplementary Table S1. Individual gene modules shown in Supplementary Figure S1C enriched for themes associated with neurogenesis (module 1), muscle/contractile (modules 2 and 3), epithelium development (module 4), and ciliation (module 5), with the top ten GO terms contained by each module shown in Supplementary Table S1. Though modules 2 and 3 enriched for muscle/contractile themes, they contained different GO term enrichment profiles in the top ten indices shown in Supplementary Table S1 and different patterns of gene activity shown in the heatmap in Figure 1C. While modules 2 and 3 were highly enriched for Muscle System Process (GO: 0003012), Muscle Contraction (GO: 0006936), and Striated Muscle Contraction (GO: 0006941), only module 2 contained genes enriched for External Encapsulating Structure (GO: 0030312) and Heart Process (GO: 0003015) shown in Supplementary Table S1.

### Transcriptome Heterogeneity Across the Developing Larynx, Esophagus, and Trachea Between E10.5 and E13.5 Is Characterized by the Diminution of Gene Activity Enriched for Neurogenesis and Cell Population Proliferation

Between E10.5 and E11.5, tracheoesophageal septation of the anterior foregut proceeds anteriorly toward the primitive LPh. It forms the respiratory diverticulum from the ventral anterior foregut endoderm and the esophagus from the dorsal anterior foregut. Prospective vocal fold (VF) cells are anteriorly positioned to the respiratory diverticulum on the ventral side of the foregut in the primitive LPh (LPh) at E10.5. The lateral walls of the primitive LPh squeeze together toward the midline, one of the first morphogenic indicators of vocal fold development. During this time, cranial neural crest cells migrate ventrally and posteriorly alongside myoblasts into the primitive LPh and anterior edges of the newly-formed esophagus and trachea. In the analysis across all tissues that included the larynx, esophagus, and trachea described above and in Methods, transcriptome variation across all three organs shows diminution between E10.5 and E13.5 in a gene module enriched for neurogenesis.

Module 1 (507 genes) in Figure 1C was highly enriched for Neuron Differentiation (GO: 0030182), Neurogenesis (GO: 0022008), and Neuron Development (GO: 0048666), shown in Supplementary Table S1, with the module profile broadly characterized by gene activity at E10.5 that is downregulated between E10.5-E13.5. Module 1 included multiple family members of neural lineage bHLH transcription factors (Neurod family) and LIM homeobox transcription factors. The largest module 2 (1,577 genes) identified in the across tissue analysis shown in Figure 1C. As described above, it was highly enriched for muscle and/or contractile themes. It also contained common genes across the primitive laryngopharynx/larynx, esophagus, and trachea enriched (90th index, p-adj 1.45E-6) for Cell Population Proliferation (GO: 0008283), including *Shh*, *Trim71*, *Bnc1*, *Lef1*, *Bex4*, and *Tert* contained by the visibly active genes shown at early timepoints in the heatmap in module 2 in Figure 1C between E10.5-E13.5.

### Transcriptome Variation Across the Larynx, Trachea, and Esophagus From E13.5-E18.5 Is Enriched for Muscle Processes, Epithelium Development, Motor-Driven Movement, and Extracellular Structure Organization

**FIGURE 2:**
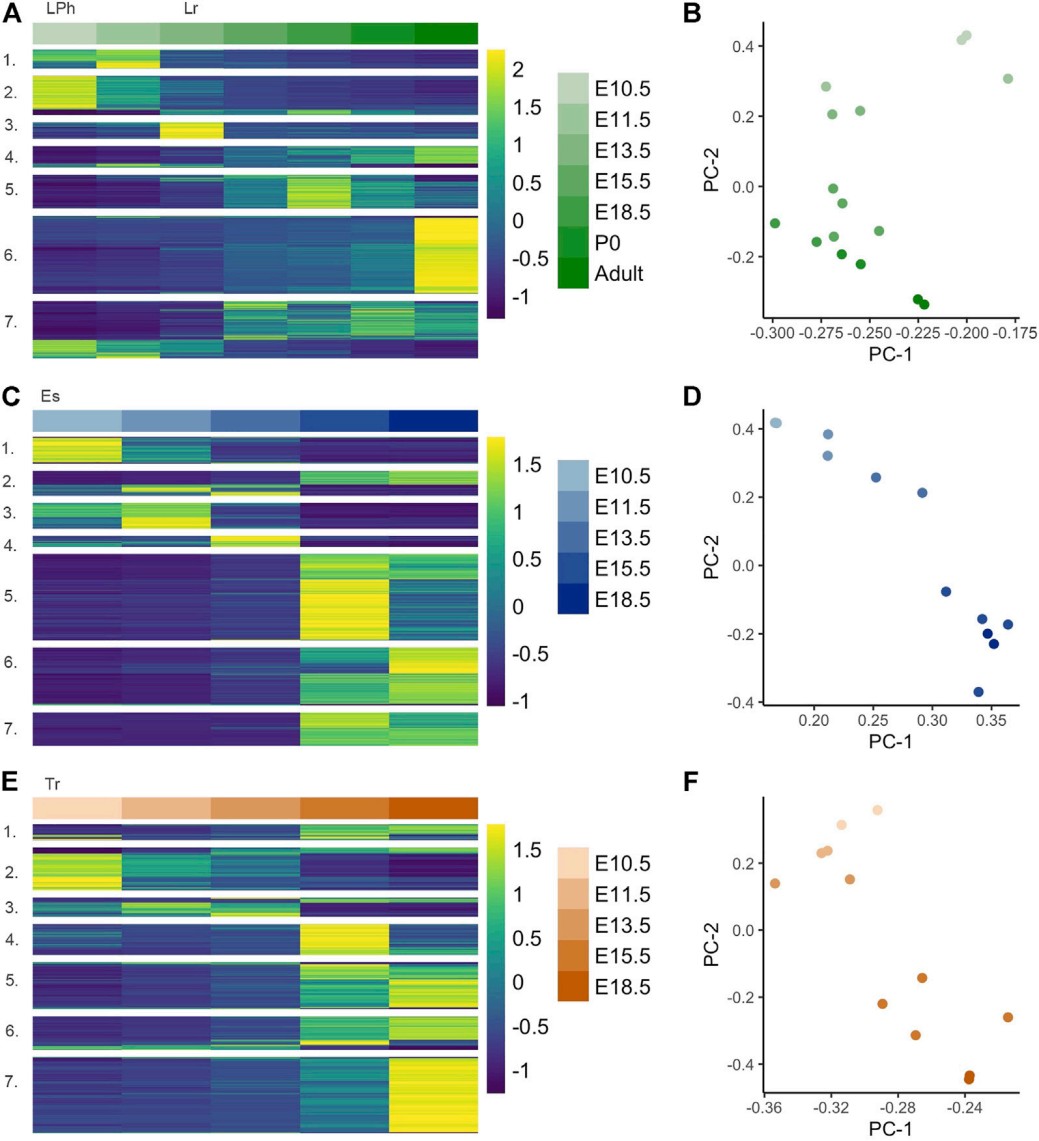
Hierarchal clustering of gene modules within developing larynx, esophagus, and trachea, **(A)** Heatmap of differentially expressed genes (DEGs) (*n* = 4,519) constructed from normalized expected counts during embryonic development of primitive laryngopharynx/larynx (LPh/Lr) across E10.5, E11.5, E13.5, E15.5, E18.5, P0, and Adult (6–8 weeks) with expression measures averaged across replicates within each tissue/age combination and then converted within each gene into a z-score for improved visual comparison; **(B)** PCA analysis demonstrates 2D representation of whole-tissue transcriptome principal components at each timepoint for the LPh/Lr; **(C)** Heatmap of DEGs in the esophagus (Es) (*n* = 2,766) across E10.5, E11.5, E13.5, E15.5, and E18.5; **(D)** PCA analysis demonstrates 2D representation of whole-tissue transcriptome principal components at each timepoint for the Es; **(E)** Heatmap of DEGs in the trachea (Tr) (*n* = 2,921) constructed from normalized expected counts during embryonic development of trachea across E10.5, E11.5, E13.5, E15.5, and E18.5; **(F)** PCA analysis demonstrates 2D representation of whole-tissue transcriptome principal components at each timepoint for the Tr.

At the level of the developing vocal folds, E13.5 is physiologically characterized by the tripartite organ process of laryngotracheo-esophageal septation. The posterior part of the larynx is ventrally continuous with the trachea and newly dorsally continuous with the laryngotracheo-esophageal septum, fully separated from the esophagus, as shown in Figure 1A (Lungova et al., 2015; Lungova and Thibeault, 2020). Laryngotracheo-esophageal septation occurs in synchrony with the initiation of EL recanalization in the larynx at E13.5, which begins to unite the ventral laryngeal cecum and dorsal pharyngoglottal duct wholly in the laryngotracheal tube. It demarks the initial realization of bilateral vocal folds and the beginning of epithelial stratification, intrinsic muscle formation and chondrification in the newly-formed larynx (Lungova et al., 2015). The muscularis externa of the murine esophagus, already developed as a smooth muscle tube, begins to transform into the striated muscle as early as E13, with the majority of esophageal striated muscle fibers developing postnatally (Patapoutian et al., 1995; Krauss et al., 2016; Zhao and Dhoot, 2000). Between E13.5 and E15.5, the esophagus’s epithelium thickens from columnar epithelium to a multi-layered transitory epithelium with a basal layer, with cardiopharyngeal mesoderm-derived progenitors and esophagus striated muscle progenitors beginning to colonize the esophagus between ∼E13-E15.5 (Rishniw et al., 2011). Tracheal epithelial cell types similarly initiate their differentiation after E13.5, when the airway smooth muscle and cartilage segmentation have been established (Kishimoto et al., 2020; Young et al., 2020). In all three tissues, epithelial differentiation is coordinated by signaling mediated by Notch, Fgf, Tgfβ, and Bmp pathways. The largest module 2 (1,577 genes) identified in the across tissue analysis described previously enriched for muscle/contractile themes, including Heart Process (GO: 0003015) and External Encapsulating Structure (GO: 0030312) (Supplementary Table S1), and included multiple Fgf and Bmp pathway members are shown in Table 1. DEGs contained by module 2, including *Fgf12*, *Tnni1*, *Tnnc1*, and *Smpx* enriched (p-adj 2.38E-11) for motifs associated with *Mef2* binding sites (Mef2_02) sourced from the Molecular Signatures Database (MSigDB), version 7.4 described in Methods. Module 2 contained multiple genes for making components of collagen subtypes, including collagen type I (*Col1a1*, *Col1a2*), collagen type III (*Col3a1*), elastin (*Eln*), and collagen component subtypes associated with cartilage development, including *Col11a2* and *Col2a1*. Module 2 contained common DEGs across the primitive laryngopharynx/larynx, esophagus, and trachea enriched (77th index, p-adj 3.97E-7) for Heart Morphogenesis (GO: 0008283), including *Mybpc3*, *Ryr2*, *Tbx20*, and *Nkx2-5*, as well as common DEGs across all tissues enriched (24th index, p-adj 1.69E-14) for Cardiac Muscle Tissue Development (GO: 0048738), including *Hnc4*, *Myocd*, *Gata5*, *Gata4*, *Tbx18*, *Tbx20*, and *Nkx2-5*. Module 3 (432 genes) was highly enriched for muscle/contractile themes (Supplementary Table S1), characterized by gene activity between E15.5-E18.5 in all tissues in Figure 1C. Module 3 included SRY-box containing gene 9 (*Sox9*) upregulated in synchrony between E13.5 and E15.5 in the larynx and trachea and upregulated between E15.5 and E18.5 in the esophagus. Module 4 (642 genes) enriched for Epithelium Development (GO: 0060429) and Epithelial Cell Differentiation (GO: 0030855), shown in Supplementary Table S1, is characterized by gene activity between E15.5-E18.5 in the larynx, esophagus, and trachea in Figure 1C, with shared genes shown in Table 1. Module 4 included Notch regulatory signaling factors *Niban2*, *Chac1*, *Llgl2*, and *Dlk2*, and Fgf regulatory factors *Tbx1*, *Zfp36*, *Esrp1*, *Esrp2*. Module 5 (314 genes) was highly enriched for ciliation, including Cilium Movement (GO: 0003341), Cilium Organization (GO: 0044782), and microtubule-based movement, with gene activity between E15.5 and E18.5, shared across all tissues(Figure 2).

**TABLE 1:** Select DEGs from top GO terms contained by the gene modules shared across the larynx, esophagus, and trachea from embryonic timepoints E10.5–E18.5 shown in Figure 1B.

Module	Theme	Genes contained by top GO terms within the module
1	Neurogenesis	*Tenm1, Brsk2, Myt1l, Wnt8b, Otp, Fzd10, Bhlhe23, Ascl1, Vxn, Neurod1, Neurod2, Neurod4, Neurod6, Spock1, Otx2, Atoh1, Fzd3, Olig3, En2, Wnt7a, Sox11, Pax6, Atp2b2, Olig2, Isl2, Lhx3, Lhx2, Lhx1, Lhx5, Lmx1b, Lhx4, Neurog1, Vsx1, Neurog2*
2	Muscle/contractile	*Mybpc3, Lef1, Ryr1, Ryr2, Tnnc1, Smpx, Tnnt1, Fgf6, Fgf2, Fgf12, Fgf7, Acta1, Myh2, Tbx18, Tbx20, Tbx22, Col1a1, Col1a2, Col3a, Col11a2, Col2a1, Nkx2-5, Nkx2-1, Nkx6-1, Myl2, Bmp3, Bmp10, Wnt3, Wnt2, Pax3, Pax8, Shh, Fgf2, Fgf6, Krt19, Hcn4, Myocd, Gata5, Gata4, Trim71, Bnc1, Bex4, Tert, Hoxa5*
3	Muscle/contractile	*Mylk2, Sox9, Csrp3, Tnnc2, Tnnt3, Tnni2, Aldoa, Scn4b, Sox21, Foxg1, Hoxb5*
4	Epithelium development	*Wnt3a, Wnt10a, Wnt4, Niban2, Chac1, Llgl2, Dlk2, Tbx1, Zfp36, Esrp1, Esrp2, Lgals3, Dlx3, Foxn1, Krt4, Tst, Spink5, Gsta1, Anxa7, Tagln2, Irf6, Ovol1, Ces1*
5	Ciliation	*Cfap61, Spag17, Spef2, Ccdc103, Dnah9, Rsph9, Cfap206, Ttc29, Cfap251, Tekt1, Zbbx, Tekt2, Tekt3, Tekt4, Spa17, Dnai1, Ccdc39, Dnai2, Rsph4a, Dnai3, Cfap73, Dnaaf1, Cfap70, Cfap91, Dnaaf6, Cfap100, Cfap54, Cfap53, Ccdc40*

The meaning of the italic values are Human ortholog gene symbols (HGNC format) mapped from the Molecular Signatures Database.

### Within-Tissue Analysis of Embryonic Larynx, Trachea, and Esophagus

Significantly varied genes within each tissue subtype across embryonic development were considered in analyses for the primitive laryngopharynx/larynx, trachea, and esophagus, with modules displayed as heatmaps shown in Figures 2A–C, respectively. The larynx analysis shown in Figure 2A included two additional timepoints for birth P0 and adulthood to characterize the influence of postnatal development and maturation on laryngeal tissue transcripts as the organ abruptly initiates protection of the lower airway and phonation at birth. Seven gene modules from analyses within the larynx, including embryo to adult timepoints [4,519 DEGs (*p* ≤ 0.01)] enriched for themes related to sensory perception/synaptic signaling (module 1, 324 genes), neurogenesis/neuron differentiation (module 2, 668 genes), cardiogenesis (module 3, 285 genes), adhesion and ion transport (module 4, 363 genes), muscle/contractile (module 5, 578 genes), epithelial cell differentiation (module 6, 1,328 genes), and ciliation (module 7, 973 genes). Each module’s top ten GO terms are shown in Supplementary Table S2. The muscle/contractile-themed module 5 across all timepoints within the larynx included multiple genes with instructions for making components of collagen subtypes. The enrichment profile for the cardiogenesis-themed module 3 found within the developing larynx shown in Supplementary Table S2 was different than muscle/contractile-themed modules described in the analysis across all three tissues, with robust enrichment for cardiomorphogenesis pathways, including Cardiac Muscle Tissue Development (GO: 0048738), Heart Process (GO: 0003015), Heart Development (GO: 0007507), and Regulation of Heart Contraction (GO: 0008016) all found within the top ten indices of the profile for the module. Across the entire laryngeal transcriptome, the P0 and Adult timepoints show visually significant upregulation (between E18.5 and P0 and between P0 and Adult) in module 6 genes, the largest module (1,328 genes) identified in this analysis, which contained the themes of enrichment terms associated with epithelial cell differentiation. Genes contained by this module were upregulated between E11.5-E13.5 and were highly active at the P0 timepoint. We found module 6 to be highly enriched (1.44E-28) for Epidermis Development (GO: 0008544) shown in Supplementary Table S2. While the Epidermis Development (GO: 0008544) term describes a process whose specific outcome is the progression of the epidermis over time, the genes contained by the term, including *Ccn2, Wnt10a, Cdsn, Lamb3, Foxn1* are in processes that result in a complex stratified squamous epithelium. Notable gene profiles across the laryngeal transcriptome with gene activity in the P0 and adult timepoints also included module 4 (363 genes) enriched for Defense Response (GO: 0002217), Cell-Cell Adhesion (GO: 0098609), and Cation Transport (GO: 0006812) with the majority of genes contained by this module characterized by gradated upregulated gene activity between E10.5 and Adult that peaks at the Adult timepoint. In the principal component analyses within the larynx shown in Figure 2B, the highest variability in gene expression across samples occurs between E10.5 and E15.5; the second-highest variability in gene expression was age-related.

The 2,766 (*p* ≤ 0.01) DEGs identified in the embryonic esophagus, clustered into seven modules shown in Figure 2C, enriched for themes related to neuron fate (module 1, 277 genes), cardiogenesis (module 2, 261 genes), synaptic signaling (module 3, 268 genes), hematopoietic processes (module 4, 114 genes), epithelial cell differentiation (module 5, 897 genes), muscle/contractile (module 6, 603 genes), and ciliation (module 7, 346 genes) shown in Supplementary Table S3. In the principal component analyses within the esophagus shown in Figure 2D, the highest and second-highest variability in gene expression across samples were age-related. The 2,921 (*p* ≤ 0.01) DEGs identified in embryonic trachea clustered into seven modules shown in Figure 2E, enriched for themes related to neuron fate specification (module 1, 176 genes), cell cycle processes (module 2, 468 genes), hematopoietic processes (module 3, 213 genes), fatty acid metabolism (module 4, 338 genes), cartilage development (module 5, 517 genes), muscle/contractile (module 6, 365 genes), and ciliation (module 7, 835 genes) shown in Supplementary Table S4. In the principal component analyses within the trachea shown in Figure 2F, the highest and second-highest variability in gene expression across samples were age-related.

## Discussion

While morphological changes of the primitive LPh/larynx, esophagus, and trachea are well described through development, transcriptome integration within and across these distinct, interdependent tissues throughout embryonic development is largely unknown. To gain insight into the expression of these interrelated gene sets, we analyzed bulk tissue RNA- seq transcriptomes within and across embryonic esophagus, trachea, and larynx through developmental time We used a gene network analysis pipeline mathematically described by WGCNA. We considered our results in the context of shared physiological processes between the three organs during development. Gene modules shared across embryonic tissues reflect the changing transcriptomes of the aerodigestive tract. The trachea, esophagus, and larynx share origin in the anterior foregut, where coordinated interactions between the definitive endoderm (DE) and the splanchnic mesoderm (SM) pattern the naïve anterior foregut tube into progenitor domains between E8.5 and E9.5 (Han et al., 2020). The DE gives rise to the epithelial lining of the larynx, esophagus, and trachea.

In contrast, the SM gives rise to the mesenchymal tissue, such as smooth and striated muscle, most cartilage, fibroblasts, and connective tissue. Han et al. recently comprehensively defined lineage diversification for the pharynx, trachea, and esophagus *via* single-cell analysis of DEGs with enriched expression in organ-specific SM and DE subtypes. Selected signatures of DEGs for E9.5 DE were *Nkx2-5/Pax1* for the pharynx, *Sox21/Klf4* for the esophagus, *Nkx2-1/Irx1* for the trachea, and DEGs with enriched expression in organ-specific E9.5 mesenchyme were *Tbx1/Irx2* for the pharynx, *Osr1/Hic1* for the esophageal, and *Sp5/Hoxa5* for respiratory mesenchyme. We found that many of these genes (*Nkx2-5*, *Sox2*1, *Nkx2-1*, *Tbx1*) and the genes that Han found to define cardiac outflow tract cells (*Nkx6-1/Gata4/Wnt2*+) were still differentially expressed in our global analysis across all tissues at later timepoints than those considered in Han’s work in modules enriched for muscle/contractile and epithelial development themes. This finding suggests a continued role for these genes in coordinating expression during the later development of embryonic muscle and epithelium in the larynx, esophagus, and trachea from E10.5 to E18.5, a window of time that captures epithelial stratification/pseudostratification events in the epithelium as well as muscle and cartilage condensation/segmentation.

Our results captured transcriptome heterogeneity in shared genes across the larynx, esophagus, and trachea at early time points E10.5-E13.5 enriched for neurogenesis. Across the temporal transcriptome of the developing embryonic larynx, trachea, and esophagus, the downregulation of highly active genes associated with neuron fate decision-making between E10.5 and E13.5 was accompanied by the downregulation of gene sets enriched for cell proliferation machinery, consistent with previous findings (Griffin et al., 2021). The upper aerodigestive system provides respiratory and alimentary functions for safe breathing and swallowing, with evidence of multiple reflexes integrating both systems in the developing fetus (Harding et al., 1984; Ross and Nijland, 1997; Miller, Sonies and Macedonia, 2003). The larynx is a highly reflexive effector organ for respiratory functions, with laryngeal afferents mediating precise monitoring of sensory events by relaying to the internal branch of the superior laryngeal nerve (iSLN) *via* dense innervation of heterologous afferent receptor subtypes (Foote and Thibeault, 2021). The laryngeal adductor response is a highly conserved sensory-afferent (iSLN) dependent reflex that results in rapid VF closure and tracheobronchial airway protection. Gene regulatory events driving the pathfinding and targeting axons in these reflexes’ embryonic larynx defy systemization. Individual adult variations in anastomoses confound histological characterization of sensory versus motor innervation and superior laryngeal nerve versus recurrent laryngeal nerve (RLN) innervation at the VFs across multiple mammalian species (Sañudo et al., 1999). The RLN controls intrinsic muscles of the larynx and relays sensory information from the mucous membranes of the larynx below the lower surface of the vocal fold, as well as sensory, secretory, and motor fibers to the cervical segments of the esophagus and the trachea.

Finding multiple differentially expressed LIM-homeodomain regulatory genes across the developing primitive laryngopharynx, trachea, and esophagus may be relevant to future regenerative endeavors requiring phenotypical characterization of developing neurons. Expression patterns of LIM-homeodomain regulatory genes by subsets of motor neurons in vertebrates constitute a combinatorial code that assigns subtype identities for motor neurons, dictates axonal pathfinding (Tsuchida et al., 1994; Sharma et al., 1998; Thor et al., 1999), and regionally parcellates the developing thalamus for sensory information relay (Nakagawa and O’Leary, 2001). LIM-homeodomain regulatory genes are expressed as progenitor cells exit the cell cycle and generate different neuron cell types in a spatiotemporal pattern. They are involved in developing thermosensory, olfactory, chemosensory, and mechanosensory neurons (Cassata et al., 2000; Sarafi-Reinach et al., 2001; Srivastava et al., 2010).

The primitive LPh, trachea, and esophagus are dorsally positioned to the developing heart loop during embryonic development. The cranial mesoderm surrounds the developing aerodigestive tract organs in the cardiopharyngeal field (CPF), a developmental domain that gives rise to the heart and craniofacial muscles (Diogo et al., 2015; Foote et al., 2019). Striated muscles in the esophagus and larynx share origins in the cranial mesoderm derived from the posterior pharyngeal arches that differentiate into branchiomeric muscle beginning at E13.5. Our data support findings that DEGs enriched for cardiogenesis processes converge on common regulatory programs found in the larynx, trachea, and esophagus (Sambasivan, Kuratani, and Tajbakhsh, 2011; Lescroart et al., 2022). Cardiocraniofacial syndromes such as DiGeorge, Velocardiofacial, CHARGE, Fetal Alcohol, Noonan syndromes, and Retinoic Acid Embryopathy are characterized by a range of comorbidities, including disruptions in the fetal development of shared organs in the aerodigestive tract with cardiac anomalies. There is a high degree of variability in disease presentation in all cases that can complicate diagnosis and treatment (Lammer et al., 1985; Bertola et al., 2004; Shprintzen, 2008; Martínez-Quintana and Rodríguez-González, 2012; Boyarchuk, Volyanska, and Dmytrash, 2017). EA, which results from incomplete separation of the ventral and dorsal parts of the foregut during development, is frequently associated with tracheoesophageal fistula and laryngeal cleft or laryngeal webbing as well as cardiac anomalies (Greenwood and Rosenthal, 1976; Diaz et al., 2005; Okuyama et al., 2006; Londahl et al., 2018). Laryngotracheo-esophageal septation at 13.5 occurs in synchrony with EL recanalization in the newly-formed larynx and intrinsic muscle formation in the VFs. This timepoint was characterized by upregulated gene activity between E11.5-E13.5 in the larynx for gene sets enriched for multiple cardiac morphogenesis pathways. They collectively modulate chamber formation, septation, and valve development in the developing heart, that is, common DEGs across the primitive laryngopharynx/larynx, esophagus, and trachea during embryonic development, the most notable of which may be *Nkx2-5*, *Gata4*, *Gata6*, *Tbx18*, *Tbx20*.

The larynx, esophagus, and trachea share multiple genes that establish ECM architecture co-expressed across and within tissues in modules enriched for muscle/contractile themes. The VFs in humans and mice are layered structures with complex multicomponent matrix composition, requiring proper patterning of these fibers for effective vibration of VF during phonation in humans or during ultrasonic acoustic signal production in rodents (Watts, Marler, and Urban, 2007; Watts, Marler and Rousseau, 2011; Johnson et al., 2010; Lungova et al., 2015; Li et al., 2016). Although the LP is present at birth as a uniform monolayer (Ishii et al., 2000; Boseley and Hartnick, 2006; Schweinfurth and Thibeault, 2008; Sato et al., 2008; Mobini, Leppik and Barker, 2016), it remains unclear what regulatory factors drive the generation of layered ECM in the LP through adulthood (Schweinfurth and Thibeault, 2008). Cell-signaling processes, including mechanically regulated positioning of fibroblasts, have been suggested to shape the formation of the LP to produce layered populations of differential cell density that, in turn, produce differential fiber compositions (Hartnick et al., 2005; Foote et al., 2019). Our study found muscle/contractile-themed DEGs common to the larynx, trachea, and esophagus enriched for regulation by a myocyte-enhancer factor (*Mef2*), an essential component of the cardiac gene regulatory network. Mef2 regulates ECM production in the mesenchymal cell lineages of the developing heart, including mechanically-induced transcriptional programs thought to drive postnatal ECM remodeling of the distinct layers in heart valves (Potthoff and Olson, 2007; Lockhart et al., 2013; Hulin et al., 2019).

Epithelium in the larynx, esophagus, and trachea is derived from the anterior foregut endoderm, where it acts as a critical boundary that defends against environmental and other stresses. In all three tissues, naïve epithelial progenitor cells derived from the anterior foregut endoderm differentiate into specialized cell types, coinciding with cell cycle slow-down after E13.5, coordinated by signaling pathways that include Wnt, Notch, Fgf, Tgfβ, and Bmp signaling. We found common Wnt, Notch, and Fgf regulatory genes across the primitive laryngopharynx, esophagus, and trachea upregulated between E13.5 and E15.5 in a gene module enriched for terms defined by processes establishing stratified squamous epithelium and multiple common Wnt, Notch, Fgf, and Bmp signaling pathway members in modules enriched for muscle/contractile themes. In the esophagus, columnar epithelium exists as a single layer of K8-expressing cells at E11.5, which starts stratifying with the reduction of K8 and K18 and gain of K5 and K14 in the basal layer at ∼E14 (Rishniw et al., 2011; Que, 2015), corresponding approximately with the initiation of stratification in the VF epithelium at E13.5 (Lungova et al., 2015), and the differentiation of epithelial progenitors in the trachea after E13.5 (Kishimoto et al., 2020). VF epithelium, which develops at the crossroad of respiratory and digestive tracts, is susceptible to repeated cycles of oscillatory collision and shearing forces during vibration, requiring a tissue-reparative response in tissue maintenance. Stratification of VF epithelium is initiated during EL recanalization at E13.5, matching when we found genes upregulated (between E11.5-E13.5) within a gene module associated with epidermal development and epithelial cell differentiation in the within-tissue analysis of the larynx. The epithelial development-themed module found in the within-tissue analysis of the larynx was most active in the adult timepoint of the mature VF epithelium. This finding suggests the transcriptional drivers initiating epithelial development are most active in the mature VF when the epithelium is characterized by rapid turnover. In addition to tolerating trauma, the characteristic of rapid turnover of the VF epithelium has been suggested to provide shape to the underlying lamina propria and is of interest to those trying to recapitulate VF mucosa (Gray, 2000).

Additionally, we found the P0 and adult timepoint across the laryngeal transcriptome to be characterized by transcriptome heterogeneity enriched for defense response and adhesion, notable for the protective role of these cell types in the context of physiological changes in the murine vocal fold after birth. After birth, amniotic fluid is replaced with air as a newborn takes its first breath, delineating the first possible encounter of the vocal fold with aerosolized foreign particles. Defense response gene sets describe processes triggered in response to the presence of a foreign body or the occurrence of an injury that restricts damage to the underlying tissue and are of interest in regenerative efforts to restore homeostasis in injured tissue. DEGs enriched for ciliation and microtubule-based movement were within and across the embryonic larynx, trachea, and esophagus with gene activity at E15.5-E18.5. The clustering of DEGs enriched for ciliation and microtubule-based processes within and across the developing larynx, esophagus, and trachea may pertain to associated ciliation events resulting from a shared mucosa origin or are motility-based ciliation required for organ expansion in other cells, or both. Numerous ciliated cells in the embryonic epithelium of the developing mouse esophagus have been described previously at E17, with these cells disappearing in histological sections by postnatal day four (Raymond, Anne, and Millane, 1991; Rodriguez et al., 2010; Rosekrans et al., 2015). The mucosa lining of the primitive laryngopharynx is continuous with the developing esophagus at earlier time points in embryonic development before the establishment of the laryngotracheo-esophageal septum at E13.5, at which time the anterior aspect of the laryngeal cavity opens into the pharynx. The posterior aspect of the laryngeal cavity is continuous with the lumen of the trachea *via* the vocal folds, which are covered by a protective layer of stratified squamous epithelium with micro ridges and microvilli on the surface that help to spread and maintain a mucous coat (Hirano et al., 1983; Lungova et al., 2015). Stratified squamous epithelium of the vocal folds transitions to a ciliated pseudostratified columnar epithelium at the supraglottis anterior to the vocal folds and infraglottis posterior to the vocal folds (Lungova et al., 2015). The epithelium lining the adult trachea is ciliated pseudostratified columnar epithelium, like that in the nasal cavity and nasopharynx.

The cartilage of the larynx interacts closely with muscle to achieve respiratory, digestive, and phonatory functions. Cranial and somatic mesoderm-derived cells give rise to the cricoid cartilage shared by the larynx and upper esophageal sphincter, arytenoid cartilages, intrinsic laryngeal muscles, and the posterior region (intermediate lamina) of the thyroid cartilage. Trachea cartilage derives from the ventral fold of lateral plate mesoderm surrounding the anterior foregut endoderm. However, spatiotemporal molecular distinctions between the more ventrally positioned respiratory mesoderm and dorsally positioned digestive mesoderm in the anterior foregut as regulatory points of origin to the cartilage in the laryngotracheal tube are unknown (Sivarao and Goyal, 2000; Plasschaert et al., 2018; Lungova and Thibeault, 2020; Kishimoto et al., 2020). In the analysis across the tissue, we found genes associated with cartilage formation, including *Sox9*, *Col11a2*, and *Col2a1*, contained within modules enriched for muscle/contractile themes and synchronously upregulated in the larynx and trachea after laryngotracheoesophageal septation between E13.5 and E15.5. Characterization of the molecular landscape in the larynx, esophagus, and trachea during anterior foregut development is a preliminary requirement for targeted stem cell, and tissue engineering approaches. Advances in understanding morphogenetic processes during larygnotracheo-esophageal septation and VF morphogenesis also provide insight into a vast category of cardiocranialfacial diseases, where whole transcription regulatory networks and downstream effectors are still unknown. A limitation of this study is the near absence of spatial information lost in bulk tissue collection processes, complicating the interpretation of gene dynamics in the anterior-posterior axis. Bulk RNA-seq uses whole tissue as a starting material, results in a mixture of different gene expression profiles, and is used to study average global gene expression, where the transcriptome programs of developing organs contain highly heterogeneous microenvironments. The true signals driving cell type differentiation can be obscured by an average gene expression profile from bulk RNA- seq, requiring future work in single-cell RNA- seq (scRNA- seq) analysis complemented by *in situ* RNA hybridization and/or spatial RNA-seq for localization of DEGs. Bulk RNA-seq analyses can provide a valuable reference point for future single-cell analysis approaches examining the regulatory relationships between genes and tracking the trajectories of distinct cell lineages in development. Characterization of the regulatory mechanisms controlling the establishment and expansion of progenitors is a necessary first step to devise future therapeutic strategies.

## Materials and Methods

### Mouse Mating and Tissue Collection

All animal experiments complied with the Public Health Service Policy on Human Care and Use of Laboratory animals and the Animal Welfare Act. The Institutional Animal Care and Use Committee of the University of Wisconsin-Madison approved the animal protocol for this investigation. Wild-type FVB/N mice, males and females, were mated. When vaginal plugs were found by noon, that day was designated as embryonic day (E) 0.5. For RNA- seq, pregnant females were sacrificed at E10.5, E11.5, E13.5, E15.5, and E18.5, mouse larynges, trachea, and esophagus were dissected, and tissues were pooled together from 10 animals at E10.5, E11.5, E13.5, and E18.5 time points, and 20 animals at the E15.5 timepoint. Approximately ∼1–3 mm pieces of tissue were excised separately from the LPh/larynx (E10.5-Adult), esophagus (E10.5-E18.5) dorsoposterior to the larynx, and trachea (E10.5-E18.5) ventroposterior to the larynx with two medial biological replicates used at each timepoint. Tissue was collected anteriorly at the level of the LPh (E10.5 and E11.5) and vocal folds (E13.5 to adult) for the larynx and immediately posterior to the larynx in the case of both the esophagus and trachea. Additional time points for postnatal stage 0 (P0) and adult (6–8 weeks) larynx were included in the “within tissue” laryngeal analysis, where bulk tissue was collected at the level of the bilateral vocal folds. All tissues were micro-dissected in an RNase-free environment and immersed in RNA later (Qiagen, Valencia, CA, United States) at 4°C overnight, and then transferred to 80°C for long-term storage before RNA extraction.

### RNA Isolation and Quality Control

RNeasy Micro Kit 50 (Qiagen, Valencia, CA, United States) was used to extract total RNA according to the manufacturer’s instructions. DNA digestion was performed using the DNase enzyme eliminator column- RNeasy MinElute spin columns. RNA concentration yield and integrity were evaluated using a Nanodrop ND-1000 spectrophotometer (Nanodrop, Wilmington, DE). Samples that met the following three criteria were retained: a concentration of 1 µg, an OD260−280 of 1.8–2.0 and an OD260:230 above two. Additionally, samples were evaluated for RNA integrity value (RIN) using the Agilent 2100 Bioanalyzer and RNA 6000 Pico kit (Agilent, Santa Clara, CA, United States) according to the manufacturer’s instructions. Only samples with electropherograms exhibiting distinct 18S and 28S ribosomal RNA (rRNA) peaks and no evidence of degradation were retained.

### RNA Sequencing

Poly-A library preparation and RNA sequencing were performed by the DNA Sequencing Facility at the University of Wisconsin-Madison Biotechnology Center per standard protocols. Briefly, first-strand cDNA using random hexamer-primed reverse transcription was generated, followed by second-strand cDNA synthesis using RNase MODULE and DNA polymerase, and ligation of sequencing adapters using the TruSeq RNA Sample Preparation Kit (Illumina, San Diego, CA, United States). Fragments of 350 bp were selected by gel electrophoresis, followed by 15 cycles of PCR amplification. The prepared libraries were sequenced on Illumina’s HiSeq 2000, and Illumina NovaSeq6000 2 × 150 S4 platforms with four RNA- seq libraries per lane with a minimum depth of 2 × 100 bp paired-end reads by the University of Wisconsin-Madison DNA Sequencing Facility in the University of Wisconsin-Madison Biotechnology Center.

### Quality Control and Gene Expression Quantification

Using standard defaults, quality control on raw sequencing files was performed *via* FastQC v011.5 (Andrews, 2010). All samples appeared high quality based on available metrics and, in particular, passed our internal quality thresholds—1) minimum per-base PHRED score of 5 and median per-base PHRED score of at least 20, and 2) modal per-sequence average PHRED score of at least 20—and were used in downstream analysis. Sequenced reads were aligned to the GRCm39 reference genome, pre-indexed files accessed from the Bowtie2 repository, using Bowtie2 (v2.4.2), accessed by RSEM (v1.3.3), which quantified observed gene transcription levels in the units of ECs from aligned reads (Li and Dewey, 2011; Langmead and Salzberg, 2012). ECs were then normalized across samples by scaling observed expression using the method of Median-Ratio implemented in the EBSeq R package. Raw and normalized expression data were remapped to human ortholog gene symbols (HGNC format) using the mapping from the Molecular Signatures Database (Mouse_ENSEMBL_Gene_ID_Human_Orthologs_MSigDB.v7.3. chip), 53,647 Ensemble genes were mapped to 18,032 human orthologs in gene symbol notation (Daley and Vere-Jones, 2003; Mootha et al., 2003; Subramanian et al., 2005). All calculations were conducted in the R environment and with Bioconductor package management (Gentleman et al., 2004; R Core Team, 2013). A tool for visualizing the expression levels of individual genes in this study is at https://data-viz.it.wisc.edu/connect/#/apps/172/access.

### Differential Expression Analysis

Genes were tested for differential expression across and within each embryonic tissue type, with the larynx-specific analysis additionally including postnatal and adult timepoints, giving four collections of significance values: one across all tissues, and one each for embryonic esophagus (e), larynx (l), and trachea (t). For this analysis, a differentially expressed gene was defined as one for which observed expression varied across time to a degree significantly different one would expect for a gene with a constant average level of expression over time. As the differences between sample times are variable, time was ranked with the earliest time point (E10.5) represented as rank 1 and the latest time point (Adult) represented as rank 7 with corresponding increments. Testing for differential expression was formally defined as testing for a significant change in average expression regressed against age rank. Testing was conducted in the Monocle2 environment (Trapnell et al., 2014) and structured as a likelihood ratio test between a natural spline regressed against age rank and a null, intercept-only model. Input data were normalized (see above section) but not log-transformed. In order to accommodate the high between-time-point variability observed during alignment QC, the error distribution was defined as Negative Binomial. Before testing, genes within tissue type were filtered, testing only those genes with at least two samples with at least a normalized expected count value of 1. Genes not passing this threshold (extremely low expressing genes) were included in the testing results with *p*-values fixed at 1. The resulting *p*-values were adjusted for multiple tests separately from the Monocle2 environment by the method of Independent Hypothesis Weighting with mean normalized expression used as the independent covariate (Ignatiadis et al., 2016; Korthauer et al., 2019).

### Module Clustering

After DE testing, significant genes (p-adj ≤ 0.01) within tissues were clustered into co-expressed groups or modules. Co-expressed genes were expected to be related to biological function, so “co-expression” was defined as a high squared correlation (Pearson type) between two genes. The squaring transformation was used as anti-correlated genes (highly negative correlation) were suspected of contributing to similar biological processes in opposite ways. Prior to the estimation of gene modules, correlation measures were transformed into robust gene-to-gene distances by the TOM implemented in the R package WGCNA (TOMdist function with option TOMType = “unsigned”) (Zhang and Horvath, 2005; Yip and Horvath, 2007; Langfelder and Horvath, 2008; Song, Langfelder and Horvath, 2012). Gene module identification was then conducted as a hierarchical clustering task (R function hclust with the method set to “ward.D”). Individual gene module identifications were produced by cutting the resulting tree at a point suggested by visual inspection.

### Enrichment Testing

As above, differential expression is defined as a significant shift in normalized expression across time points. Gene sets enriched for DEGs therefore denoted functional groups whose activity likewise changed over the developmental time course. Enrichment testing was conducted across all tissue types, clustered gene modules, and gene-set collections for GO terms (GO, biological process sub-collection) and Transcription Factors. All references relating individual genes to curated gene sets were sourced from the Molecular Signatures Database (MSigDB), version 7.4, and were defined in terms of human ortholog gene symbols (Ashburner et al., 2000; Kanehisa and Goto, 2000; Liberzon et al., 2015; Kanehisa et al., 2019; Yevshin et al., 2019; ‘The Gene Ontology resource: enriching a GOld mine. Consortium’, 2021; Kanehisa, Sato and Kawashima, 2021). Testing was conducted in the R piano environment (Väremo, Nielsen, and Nookaew, 2013). Enrichment within individual gene co-expression modules was performed on module membership (coded as 0/1) as a binary hypergeometric test again against the full set of 18,032 genes. Adjusted *p*-values were calculated internally by the runGSA function in all cases.

## Data Availability

The data presented in this study are deposited in the GEO repository accession number GSE206673.
