# Supplementary material for: Transcriptome Dynamics in the Developing Larynx, Trachea, and Esophagus
Source: Front Cell Dev Biol. 2022 Jul 22;10:942622. doi: 10.3389/fcell.2022.942622 (PMC9353518; doi:10.3389/fcell.2022.942622)
Supplement: Supplementary file 1 [file Table1.docx]

Supplementary Material

**Supplementary Table S1
GO Biological Process Enrichment across All Embryonic Tissues**

GO Biological Process Enrichment for Module 1 (507 genes)

| **Index** | **GO Term** | **p** | **pAdj** |
| --- | --- | --- | --- |
| **1** | **Neuron Differentiation** | 1.3556146415443e-52 | 1.01399975187514e-48 |
| **2** | **Neurogenesis** | 1.1193902567304e-50 | 4.1865195601717e-47 |
| **3** | **Central Nervous System Development** | 5.62357769343994e-39 | 1.40214537156436e-35 |
| **4** | **Neuron Development** | 1.54965331944117e-37 | 2.89785170735498e-34 |
| **5** | **Cell Part Morphogenesis** | 2.49469104897174e-29 | 3.73205780926173e-26 |
| **6** | **Cell Morphogenesis Involved in Neuron Differentiation** | 1.44442638877801e-28 | 1.80071823134325e-25 |
| **7** | **Head Development** | 1.97513014232295e-28 | 2.11056763779652e-25 |
| **8** | **Cellular Component Morphogenesis** | 2.14624642577008e-26 | 2.00674040809502e-23 |
| **9** | **Axon Development** | 5.55895937404562e-26 | 4.62011290198458e-23 |
| **10** | **Synaptic Signaling** | 8.61144752027032e-25 | 6.4413627451622e-22 |

GO Biological Process Enrichment for Module 2 (1577 genes)

| **Index** | **GO Term** | **p** | **pAdj** |
| --- | --- | --- | --- |
| 1 | **Muscle System Process** | 1.99345352888943e-36 | 1.49110323960929e-32 |
| 2 | **Muscle Contraction** | 2.00301224630437e-35 | 7.49126580117833e-32 |
| 3 | **External Encapsulating Structure** | 3.67970635914403e-35 | 9.17473452213244e-32 |
| 4 | **Muscle Structure Development** | 9.32888496303719e-30 | 1.74450148808795e-26 |
| 5 | **Circulatory System Process** | 1.56491073132338e-28 | 2.34110645405978e-25 |
| 6 | **Muscle Tissue Development** | 1.3352996808901e-27 | 1.66467360217633e-24 |
| 7 | **Striated Muscle Cell Differentiation** | 2.18098523504098e-27 | 2.33053850830093e-24 |
| 8 | **Striated Muscle Contraction** | 6.45355968488811e-27 | 6.03407830537038e-24 |
| 9 | **Heart Process** | 7.661707145045E-26 | 6.36772993832629e-23 |
| 10 | **Muscle Cell Development** | 1.35138230165471e-25 | 1.01083396163772e-22 |

GO Biological Process Enrichment for Module 3 (432 genes)

| **Index** | **GO Term** | **p** | **pAdj** |
| --- | --- | --- | --- |
| **1** | **Muscle System Process** | 1.08155239567562e-13 | 8.09001191965363e-10 |
| **2** | **Muscle Contraction** | 1.0303338567788e-11 | 3.85344862435272e-08 |
| **3** | **Regulation of System Process** | 1.47862698434415e-10 | 3.68670994763141e-07 |
| **4** | **Striated Muscle Contraction** | 8.03672833043124e-10 | 1.50286819779064e-06 |
| **5** | **Circulatory System ProcessG** | 4.04932327585252e-08 | 6.05778762067537e-05 |
| **6** | **Cation Transport** | 1.37994260733712e-07 | 0.000172032845048028 |
| **7** | **Regulation of Muscle System Process** | 2.31826445503286e-07 | 0.000247723116052083 |
| **8** | **Inorganic Ion Transmembrane Transport** | 3.32956197748234e-07 | 0.000311314044894598 |
| **9** | **Epidermis Development** | 5.32407773216835e-07 | 0.000442490015962436 |
| **10** | **Regulation of Peptidase Activity** | 9.23148397341784e-07 | 0.000690515001211654 |

GO Biological Process Enrichment for Module 4 (642 genes)

| **Index** | **GO Term** | **p** | **pAdj** |
| --- | --- | --- | --- |
| **1** | **Epidermis Development** | 1.14803976039255e-35 | 8.58733740773626e-32 |
| **2** | **Keratinocyte Differentiation** | 4.12471683450716e-33 | 1.51316574455072e-29 |
| **3** | **Keratinization** | 6.06884656905368e-33 | 1.51316574455072e-29 |
| **4** | **Epidermal Cell Differentiation** | 6.31948668670241e-32 | 1.18174401041335e-28 |
| **5** | **Skin Development** | 1.42963738885771e-31 | 2.13873753373113e-28 |
| **6** | **Cornification** | 5.73155326099561e-30 | 7.14533639870786e-27 |
| **7** | **Epithelial Cell Differentiation** | 4.15516999127505e-21 | 4.44009593353391e-18 |
| **8** | **Epithelium Development** | 3.21126293396379e-13 | 3.00253084325615e-10 |
| **9** | **Unsaturated Fatty Acid Metabolic Process** | 9.7121514457801e-13 | 8.07187697938168e-10 |
| **10** | **Icosanoid Metabolic Process** | 2.29663040494796e-12 | 1.71787954290108e-09 |

GO Biological Process enrichment for Module 5 (314 genes)

| **Index** | **GO Term** | **p** | **pAdj** |
| --- | --- | --- | --- |
| **1** | **Cilium Movement** | 2.95263912649164e-63 | 2.20857406661575e-59 |
| **2** | **Cilium Organization** | 7.22846541578997e-56 | 2.70344606550545e-52 |
| **3** | **Axoneme Assembly** | 2.13106043340535e-54 | 5.31344401395734e-51 |
| **4** | **Microtubule Based Movement** | 9.11038068545968e-51 | 1.70364118818096e-47 |
| **5** | **Microtubule Bundle Formation** | 1.62212440896404e-49 | 2.4266981158102e-46 |
| **6** | **Microtubule Based Process** | 2.32598080714005e-42 | 2.89972273956793e-39 |
| **7** | **Cell Projection Assembly** | 1.65933688513666e-37 | 1.77311998583174e-34 |
| **8** | **Cilium or Flagellum Dependent Cell Motility** | 2.30345316054314e-37 | 2.15372870510784e-34 |
| **9** | **Organelle Assembly** | 1.33572879953025e-31 | 1.11013904672069e-28 |
| **10** | **Microtubule Cytoskeleton Organization** | 3.61731157475721e-27 | 2.70574905791839e-24 |

**Supplementary Table S2**

**GO Biological Process Enrichment within Larynx from E10.5 to Adult**

GO Biological Process Enrichment for Module 1 (324 genes)

| **Index** | **GO Term** | **p** | **pAdj** |
| --- | --- | --- | --- |
| **1** | **Nervous System Process** | 2.19047694251432e-28 | 1.63847675300071e-24 |
| **2** | **Sensory Perception** | 3.69716171092097e-18 | 1.38273847988444e-14 |
| **3** | **Synaptic Signaling** | 7.51580483548545e-18 | 1.87394067231437e-14 |
| **4** | **Detection of Stimulus Involved In Sensory Perception** | 1.85170631956278e-16 | 3.4626908175824e-13 |
| **5** | **Sensory Perception of Chemical Stimulus** | 1.82688510688862e-15 | 2.68951006898021e-12 |
| **6** | **Synapse Organization** | 2.15736101789856e-15 | 2.68951006898021e-12 |
| **7** | **Sensory Perception of Smell** | 4.34667529561286e-15 | 4.64473303016917e-12 |
| **8** | **Detection of Chemical Stimulus** | 2.84733878004648e-14 | 2.66226175934346e-11 |
| **9** | **Neuron Differentiation** | 1.31233336233975e-13 | 1.09069483892237e-10 |
| **10** | **Neurogenesis** | 1.61045779823406e-13 | 1.20462243307908e-10 |

GO Biological Process Enrichment for Module 2 (668 genes)

| Index | GO Term | p | pAdj |
| --- | --- | --- | --- |
| 1 | Neurogenesis | 4.79881890941834e-28 | 1.8471571164885e-24 |
| 2 | Neuron Differentiation | 4.93892277135962e-28 | 1.8471571164885e-24 |
| 3 | Central Nervous System Development | 9.10628425124238e-25 | 2.2705002066431e-21 |
| 4 | Head Development | 3.11219345593101e-23 | 5.819801762591E-20 |
| 5 | Cell Cycle | 5.91120029431608e-20 | 8.84315564029686e-17 |
| 6 | Cell Part Morphogenesis | 1.68446819019231e-19 | 2.09997034377308e-16 |
| 7 | Forebrain Development | 3.31511847677797e-19 | 3.0996357757874e-16 |
| 8 | Cell Morphogenesis Involved in Neuron Differentiation | 2.95833046668607e-19 | 3.0996357757874e-16 |
| 9 | Neuron Development | 3.56170548362065e-18 | 2.96017300194249e-15 |
| 10 | Axon Development | 4.29460376024246e-18 | 3.21236361266136e-15 |

GO Biological Process Enrichment for Module 3 (285 genes)

| **Index** | **GO Term** | **p** | **pAdj** |
| --- | --- | --- | --- |
| **1** | **Circulatory System Process** | 1.21766626593588e-28 | 9.10814366920037e-25 |
| **2** | **Regulation of System Process** | 9.84869713383401e-26 | 3.68341272805392e-22 |
| **3** | **Cardiac Muscle Tissue Development** | 6.40465008319753e-24 | 1.59689275407725e-20 |
| **4** | **Heart Process** | 2.46291362579058e-22 | 3.68451878418271e-19 |
| **5** | **Regulation of Blood Circulation** | 2.46291362579058e-22 | 3.68451878418271e-19 |
| **6** | **Muscle Contraction** | 1.8274822857704e-21 | 1.70869593719533e-18 |
| **7** | **Heart Development** | 1.51387599995484e-21 | 1.70869593719533e-18 |
| **8** | **Cardiac Muscle Contraction** | 1.68705205030858e-21 | 1.70869593719533e-18 |
| **9** | **Regulation of Heart Contraction** | 1.24218237176239e-20 | 1.03239157119807e-17 |
| **10** | **Muscle Tissue Development** | 1.98785774057228e-19 | 1.48691758994807e-16 |

GO Biological Process Enrichment for Module 4 (363 genes)

| **Index** | **GO Term** | **p** | **pAdj** |
| --- | --- | --- | --- |
| **1** | **Defense Response** | 4.47018928509579e-08 | 0.000334370158525165 |
| **2** | **Biological Adhesion** | 4.14267417032365e-06 | 0.0154936013970104 |
| **3** | **Cell Cell Adhesion** | 7.34812610610844e-06 | 0.0183213277578971 |
| **4** | **Negative Regulation of Cellular Response to Insulin Stimulus** | 1.11657958941532e-05 | 0.0208800383220665 |
| **5** | **Cation Transport** | 1.68841922813783e-05 | 0.025258751652942 |
| **6** | **Inflammatory Response** | 2.95207671987305e-05 | 0.0262684161218642 |
| **7** | **Response to Cold** | 3.44290523346821e-05 | 0.0262684161218642 |
| **8** | **Response to Biotic Stimulus** | 2.56675717042074e-05 | 0.0262684161218642 |
| **9** | **Response to Endogenous Stimulus** | 2.32497269324884e-05 | 0.0262684161218642 |
| **10** | **Inorganic Ion Transmembrane Transport** | 3.51182033714762e-05 | 0.0262684161218642 |

GO Biological Process Enrichment for Module 5 (578 genes)

| **Index** | **GO Term** | **p** | **pAdj** |
| --- | --- | --- | --- |
| **1** | **Muscle System Process** | 1.1019518785531e-37 | 8.24260005157721e-34 |
| **2** | **Muscle Contraction** | 1.00953534802327e-34 | 3.77566220160704e-31 |
| **3** | **Muscle Structure Development** | 4.4096399672513e-34 | 1.09947023183466e-30 |
| **4** | **Muscle Filament Sliding** | 2.64202755651281e-28 | 4.94059153067895e-25 |
| **5** | **Muscle Organ Development** | 9.01922046291158e-28 | 1.34927538125157e-24 |
| **6** | **Striated Muscle Cell Development** | 9.35362037161714e-27 | 1.16608467299494e-23 |
| **7** | **Muscle Cell Development** | 7.83931264084341e-25 | 8.3768655076441e-22 |
| **8** | **Myofibril Assembly** | 1.20539880635593e-23 | 1.1270478839428e-20 |
| **9** | **Muscle Cell Differentiation** | 2.62086402061524e-23 | 2.17822920824467e-20 |
| **10** | **External Encapsulating Structure Organization** | 5.77025964381466e-23 | 4.31615421357337e-20 |

GO Biological Process Enrichment for Module 6 (1328 genes)

| **Index** |  | **p** | **pAdj** |
| --- | --- | --- | --- |
| **1** | **Epidermis Development** | 1.92554412993056e-32 | 1.44030700918806e-28 |
| **2** | **Keratinocyte Differentiation** | 5.11976870939164e-30 | 1.33202136283438e-26 |
| **3** | **Cornification** | 5.34233166912183e-30 | 1.33202136283438e-26 |
| **4** | **Epidermal Cell Differentiation** | 2.11143185261456e-29 | 3.94837756438923e-26 |
| **5** | **Keratinization** | 1.2175852426294e-28 | 1.82150752297358e-25 |
| **6** | **Skin Development** | 3.0590720147996e-28 | 3.81364311178351e-25 |
| **7** | **Defense Response** | 5.72139076234526e-26 | 6.11371470033465e-23 |
| **8** | **Epithelial Cell Differentiation** | 4.03358019329952e-23 | 3.77139748073506e-20 |
| **9** | **Defense Response to Other Organism** | 4.18955059170521e-22 | 3.48198204732833e-19 |
| **10** | **Defense Response To Biotic Stimulus** | 1.42969453733766e-21 | 1.06941151392857e-18 |

GO Biological Process Enrichment for Module 7 (973 genes)

| **Index** | **GO Term** | **p** | **pAdj** |
| --- | --- | --- | --- |
| **1** | **Cilium Movement** | 4.30277665970219e-19 | 1.65560889650755e-15 |
| **2** | **Microtubule Based Process** | 4.42676175536777e-19 | 1.65560889650755e-15 |
| **3** | **Microtubule Bundle Formation** | 9.31894467684363e-18 | 2.32352353942634e-14 |
| **4** | **Axoneme Assembly** | 8.91828412886594e-17 | 1.66771913209793e-13 |
| **5** | **Microtubule Cytoskeleton Organization** | 1.19546301956906e-15 | 1.78841267727531e-12 |
| **6** | **Cilium or Flagellum Dependent Cell Motility** | 3.83157715428044e-14 | 4.77669951900295e-11 |
| **7** | **Microtubule Based Movement** | 7.36686139844914e-14 | 7.87201760862852e-11 |
| **8** | **Cilium Organization** | 7.3060795611869e-13 | 6.83118438970975e-10 |
| **9** | **External Encapsulating Structure Organization** | 1.43801041912849e-10 | 1.19514643723124e-07 |
| **10** | **DNA Packaging** | 2.19536256730335e-08 | 1.64213120034291e-05 |

**Supplementary Table S3**

**GO Biological Process Enrichment within Embryonic Esophagus**

GO Biological Process Enrichment for Module 1 (277 genes)

| **Index** | **GO Term** | **p** | **pAdj** |
| --- | --- | --- | --- |
| **1** | **Coagulation** | 1.74913146153667e-13 | 1.30835033322943e-09 |
| **2** | **Regulation of Coagulation** | 7.34373281887889e-13 | 2.15438446387566e-09 |
| **3** | **Negative Regulation of Coagulation** | 8.64057940057082e-13 | 2.15438446387566e-09 |
| **4** | **Fibrinolysis** | 1.96715831007696e-12 | 3.67858603984392e-09 |
| **5** | **Protein Activation Cascade** | 5.5648255619563e-12 | 8.32497904068663e-09 |
| **6** | **Negative Regulation of Peptidase Activity** | 1.21286955485451e-11 | 1.46204952836243e-08 |
| **7** | **Negative Regulation of Hydrolase Activity** | 1.36822816825361e-11 | 1.46204952836243e-08 |
| **8** | **Regulation of Body Fluid Levels** | 1.700443557219E-11 | 1.58991472599976e-08 |
| **9** | **Negative Regulation of Wound Healing** | 2.28199975459096e-11 | 1.89659535159338e-08 |
| **10** | **Wound Healing** | 5.27548616678084e-11 | 3.94606365275207e-08 |

GO Biological Process Enrichment for Module 2 (261 genes)

| **Index** | **GO Term** | P | pAdj |
| --- | --- | --- | --- |
| **1** | **Muscle System Process** | 1.4896299197468e-15 | 5.57121589985304e-12 |
| **2** | **Regulation of System Process** | 7.94617300103175e-16 | 5.57121589985304e-12 |
| **3** | **Muscle Contraction** | 1.69008359734591e-14 | 4.21394176938247e-11 |
| **4** | **Striated Muscle Contraction** | 5.75940438568469e-14 | 1.07700862012304e-10 |
| **5** | **Circulatory System Process** | 3.2364689194257e-13 | 4.84175750346085e-10 |
| **6** | **Regulation of Striated Muscle Contraction** | 1.7820693816491e-12 | 2.22164649578921e-09 |
| **7** | **Heart Process** | 3.67876932310844e-12 | 3.43964931710639e-09 |
| **8** | **Regulation of Blood Circulation** | 3.67876932310844e-12 | 3.43964931710639e-09 |
| **9** | **Regulation of Heart Contraction** | 5.98318936050944e-12 | 4.51863103711603e-09 |
| **10** | **Cardiac Muscle Contraction** | 6.04095058437972e-12 | 4.51863103711603e-09 |

GO Biological Process Enrichment for Module 3 (268 genes)

| **Index** |  | **p** | **pAdj** |
| --- | --- | --- | --- |
| **1** | **Synaptic Signaling** | 4.0772247051736e-30 | 3.04976407946986e-26 |
| **2** | **Neuron Differentiation** | 8.15950867600126e-24 | 3.05165624482447e-20 |
| **3** | **Neurogenesis** | 1.499678301498E-22 | 3.73919789840169e-19 |
| **4** | **Neuron Development** | 9.8849337060358e-22 | 1.84848260302869e-18 |
| **5** | **Cell Cell Signaling** | 4.10107035515559e-20 | 6.13520125131276e-17 |
| **6** | **Cell Part Morphogenesis** | 4.71119221175397e-19 | 5.87328629065328e-16 |
| **7** | **Synapse Organization** | 3.41357897795538e-17 | 3.64765296501518e-14 |
| **8** | **Cellular Component Morphogenesis** | 5.8790053742575e-17 | 5.49687002493076e-14 |
| **9** | **Behavior** | 7.34350296233183e-16 | 6.10326690647135e-13 |
| **10** | **Cell Morphogenesis Involved In Neuron Differentiation** | 1.9666418971448e-15 | 1.47104813906431e-12 |

GO Biological Process Enrichment for Module 4 (114 genes)

| **Index** |  | **p** | **pAdj** |
| --- | --- | --- | --- |
| **1** | **Coagulation** | 1.74913146153667e-13 | 1.30835033322943e-09 |
| **2** | **Regulation of Coagulation** | 7.34373281887889e-13 | 2.15438446387566e-09 |
| **3** | **Negative Regulation of Coagulation** | 8.64057940057082e-13 | 2.15438446387566e-09 |
| **4** | **Fibrinolysis** | 1.96715831007696e-12 | 3.67858603984392e-09 |
| **5** | **Protein Activation Cascade** | 5.5648255619563e-12 | 8.32497904068663e-09 |
| **6** | **Regulation of Peptidase Activity** | 1.21286955485451e-11 | 1.46204952836243e-08 |
| **7** | **Negative Regulation of Hydrolase Activity** | 1.36822816825361e-11 | 1.46204952836243e-08 |
| **8** | **Regulation of Body Fluid Levels** | 1.700443557219E-11 | 1.58991472599976e-08 |
| **9** | **Negative Regulation of Wound Healing** | 2.28199975459096e-11 | 1.89659535159338e-08 |
| **10** | **Wound Healing** | 5.27548616678084e-11 | 3.94606365275207e-08 |

GO Biological Process Enrichment for Module 5 (897 genes)

| **Index** |  | **p** | **pAdj** |
| --- | --- | --- | --- |
| **1** | **Keratinization** | 1.13575309771164e-34 | 8.49543317088309e-31 |
| **2** | **Cornification** | 3.68529839117633e-34 | 1.37830159829995e-30 |
| **3** | **Epidermis Development** | 1.07362164845622e-32 | 2.67689664348418e-29 |
| **4** | **Keratinocyte Differentiation** | 9.427502411539E-32 | 1.76294295095779e-28 |
| **5** | **Epidermal Cell Differentiation** | 1.67795263295505e-29 | 2.51021713890076e-26 |
| **6** | **Skin Development** | 4.8380657028871e-29 | 6.03145524293258e-26 |
| **7** | **Epithelial Cell Differentiation** | 2.20893353830541e-16 | 2.36040326664636e-13 |
| **8** | **Defense Response** | 5.01093194165165e-13 | 4.6852213654443e-10 |
| **9** | **Defense Response to Other Organism** | 9.01776461210781e-13 | 7.49476436650738e-10 |
| **10** | **Icosanoid Metabolic Process** | 8.08037594076198e-12 | 6.04412120368996e-09 |

GO Biological Process Enrichment for Module 6 (603 genes)

| **Index** |  | **p** | **pAdj** |
| --- | --- | --- | --- |
| **1** | **Muscle System Process** | 1.57004314948006e-23 | 1.17439227581109e-19 |
| **2** | **Muscle Contraction** | 5.16044169187117e-21 | 1.93000519275982e-17 |
| **3** | **Striated Muscle Cell Development** | 2.2352405260457e-20 | 5.57319971160729e-17 |
| **4** | **Muscle Structure Development** | 1.50994255347136e-19 | 2.82359257499145e-16 |
| **5** | **Muscle Filament Sliding** | 2.55614688308824e-18 | 3.8239957371E-15 |
| **6** | **Myofibril Assembly** | 6.60916459924332e-18 | 8.23942520039E-15 |
| **7** | **Muscle Cell Development** | 1.74343644398852e-17 | 1.8629863715763e-14 |
| **8** | **Striated Muscle Cell Differentiation** | 3.4300933132195e-17 | 3.20713724786024e-14 |
| **9** | **External Encapsulating Structure Organization** | 4.61276915672675e-17 | 3.83372369914623e-14 |
| **10** | **Muscle Organ Development** | 3.74011059330118e-15 | 2.79760272378928e-12 |

GO Biological Process Enrichment for Module 7 (346 genes)

| **Index** | **GO Term** | **p** | **pAdj** |
| --- | --- | --- | --- |
| **1** | **Cilium movement** | 9.6296123208216e-64 | 7.20295001597456e-60 |
| **2** | **Axoneme assembly** | 3.31820725733496e-58 | 1.24100951424328e-54 |
| **3** | **Microtubule bundle formation** | 7.76670121857108e-51 | 1.93649750383039e-47 |
| **4** | **Cilium organization** | 3.17401664592777e-50 | 5.93541112788492e-47 |
| **5** | **Microtubule based movement** | 5.85460182352261e-49 | 8.75848432798982e-46 |
| **6** | **Microtubule based process** | 1.09427699218395e-39 | 1.36419865025599e-36 |
| **7** | **Cilium or flagellum dependent cell motility** | 3.73706400208157e-37 | 3.99331981936716e-34 |
| **8** | **Cell projection assembly** | 9.59933259839005e-34 | 8.97537597949469e-31 |
| **9** | **Axonemal dynein complex assembly** | 1.05242804038135e-31 | 8.74684638005834e-29 |
| **10** | **Organelle assembly** | 6.459411041239E-28 | 4.83163945884677e-25 |

**Supplementary Table S4**

**GO Biological Process Enrichment Within Embryonic Trachea**

GO Biological Enrichment for Module 1 (176 genes)

| **Index** | **GO Term** | **p** | **pAdj** |
| --- | --- | --- | --- |
| **1** | **Ossification** | 1.14916435099985e-08 | 8.59574934547888e-05 |
| **2** | **Neurogenesis** | 5.54653095179654e-07 | 0.00207440257597191 |
| **3** | **Neuron Differentiation** | 2.08218727776149e-06 | 0.00519158694588531 |
| **4** | **Positive Regulation Of Multicellular Organismal Process** | 6.76206815487851e-06 | 0.0126450674496228 |
| **5** | **Bone Mineralization** | 1.31908616373837e-05 | 0.0139749464515627 |
| **6** | **Secretion** | 1.49464667931152e-05 | 0.0139749464515627 |
| **7** | **Synapse Organization** | 1.04198748037708e-05 | 0.0139749464515627 |
| **8** | **Positive Regulation Of Developmental Process** | 1.17766016917976e-05 | 0.0139749464515627 |
| **9** | **Central Nervous System Development** | 2.03610265768467e-05 | 0.0163168289475256 |
| **10** | **Neuron Development** | 2.39953366875376e-05 | 0.0163168289475256 |

GO Biological Process Enrichment for Module 2 (468 genes)

| **Index** | GO Term | **p** | **pAdj** |
| --- | --- | --- | --- |
| **1** | **Cell Cycle** | 2.87480826451267e-32 | 2.15035658185548e-28 |
| **2** | **Mitotic Cell Cycle** | 2.01382086054196e-29 | 7.53169001842693e-26 |
| **3** | **Chromosome Organization** | 6.49259710997158e-27 | 1.61882087941958e-23 |
| **4** | **Cell Cycle Process** | 1.00756117587564e-26 | 1.88413939888744e-23 |
| **5** | **Cell Division** | 9.15433910799301e-26 | 1.36948913055575e-22 |
| **6** | **Dna Replication** | 2.59524216338604e-23 | 3.23540189702126e-20 |
| **7** | **Chromosome Segregation** | 2.40476773775834e-21 | 2.56966609691891e-18 |
| **8** | **Mitotic Sister Chromatid Segregation** | 9.22426433399622e-20 | 8.62468715228646e-17 |
| **9** | **Sister Chromatid Segregation** | 2.33249985133143e-19 | 1.93856654310657e-16 |
| **10** | **Mitotic Nuclear Division** | 5.74160194581364e-19 | 4.2947182554686e-16 |

GO Biological Process Enrichment for Module 3 (213 genes)

| **Index** | **GO Term** | **p** | **pAdj** |
| --- | --- | --- | --- |
| **1** | **Coagulation** | 1.28952272757301e-14 | 9.64563000224609e-11 |
| **2** | **Regulation Of Body Fluid Levels** | 6.18906474626812e-14 | 2.31471021510428e-10 |
| **3** | **Protein Activation Cascade** | 4.66544602474854e-13 | 1.1632512088373e-09 |
| **4** | **Negative Regulation Of Coagulation** | 7.37012516591766e-13 | 1.3782134060266e-09 |
| **5** | **Response To Wounding** | 2.31838730488895e-12 | 2.16769213007116e-09 |
| **6** | **Wound Healing** | 1.91129225450461e-12 | 2.16769213007116e-09 |
| **7** | **Regulation Of Coagulation** | 1.91988079027692e-12 | 2.16769213007116e-09 |
| **8** | **Negative Regulation Of Multicellular Organismal Process** | 2.07950210736414e-12 | 2.16769213007116e-09 |
| **9** | **Fibrinolysis** | 6.55591290642626e-12 | 5.4486920600076e-09 |
| **10** | **Cardiac Muscle Tissue Development** | 3.81969131070279e-11 | 2.5973900912779e-08 |

GO Biological Process Enrichment for Module 4 (338 genes)

| **Index** | **GO Term** | **p** | **pAdj** |
| --- | --- | --- | --- |
| **1** | **Synaptic Signaling** | 1.59766037993116e-27 | 1.19504996418851e-23 |
| **2** | **Neurotransmitter Transport** | 2.99666423038551e-20 | 1.12075242216418e-16 |
| **3** | **Lipid Catabolic Process** | 6.54503349345051e-18 | 1.63189501770033e-14 |
| **4** | **Cellular Lipid Catabolic Process** | 2.57023566561817e-17 | 4.80634069470598e-14 |
| **5** | **Cell Cell Signaling** | 1.50586388758104e-16 | 2.25277237582124e-13 |
| **6** | **Fatty Acid Metabolic Process** | 5.19058095395832e-16 | 6.4709242559347e-13 |
| **7** | **Organic Acid Metabolic Process** | 9.50544466339822e-16 | 1.01572465831741e-12 |
| **8** | **Fatty Acid Beta Oxidation** | 1.88653479469069e-15 | 1.76391003303579e-12 |
| **9** | **Regulation Of Neurotransmitter Levels** | 3.00995650231268e-15 | 2.04677042157262e-12 |
| **10** | **Cation Transport** | 2.79933821831345e-15 | 2.04677042157262e-12 |

GO Biological Process Enrichment for Module 5 (517 genes)

| **Index** | **GO Term** | **p** | **pAdj** |
| --- | --- | --- | --- |
| **1** | **External Encapsulating Structure Organization** | 1.89671118335459e-21 | 1.41873996514923e-17 |
| **2** | **Skeletal System Development** | 1.58007888576135e-13 | 5.90949503274746e-10 |
| **3** | **Cartilage Development** | 2.68182991331619e-12 | 5.01502193790127e-09 |
| **4** | **Connective Tissue Development** | 2.36468178372104e-12 | 5.01502193790127e-09 |
| **5** | **Thyroid Hormone Metabolic Process** | 1.21049574460254e-09 | 1.81090163392541e-06 |
| **6** | **Thyroid Hormone Generation** | 2.64225254655682e-09 | 3.29400817470751e-06 |
| **7** | **Chondrocyte Differentiation** | 4.65014653345096e-09 | 4.96901372431617e-06 |
| **8** | **Collagen Fibril Organization** | 2.48683743774E-08 | 2.3251930042869e-05 |
| **9** | **Ossification** | 4.3884312089924e-07 | 0.000364727393814035 |
| **10** | **Circulatory System Process** | 5.44918854141042e-07 | 0.000407599302897499 |

Go Biological Process Enrichment for Module 6 (365 genes)

| **Index** |  | **p** | **pAdj** |
| --- | --- | --- | --- |
| **1** | **Muscle System Process** | 1.49392743017951e-22 | 1.11745771777427e-18 |
| **2** | **Muscle Contraction** | 2.92556317904894e-20 | 1.0941606289643e-16 |
| **3** | **Muscle Filament Sliding** | 3.91134141369055e-13 | 9.75227792480176e-10 |
| **4** | **Striated Muscle Contraction** | 4.80841111523131e-12 | 8.99172878548255e-09 |
| **5** | **Muscle Structure Development** | 8.99478849049117e-12 | 1.34562035817748e-08 |
| **6** | **Actin Mediated Cell Contraction** | 3.08261860773668e-11 | 3.84299786431173e-08 |
| **7** | **T Cell Differentiation** | 1.2940765389302e-10 | 1.38046137907772e-07 |
| **8** | **T Cell Activation** | 1.47642928243606e-10 | 1.38046137907772e-07 |
| **9** | **Antigen Receptor Mediated Signaling Pathway** | 1.98509890563016e-10 | 1.64983775712373e-07 |
| **10** | **Adaptive Immune Response** | 1.36308713170485e-09 | 9.26899249559295e-07 |

GO Biological Process Enrichment for Module 7 (835 genes)

| **Index** | **GO Term** | **p** | **pAdj** |
| --- | --- | --- | --- |
| **1** | **Cilium Movement** | 2.56911878224481e-27 | 1.92170084911912e-23 |
| **2** | **Axoneme Assembly** | 1.58770942216942e-25 | 5.93803323891365e-22 |
| **3** | **Microtubule Bundle Formation** | 7.44367235979665e-22 | 1.8559556417093e-18 |
| **4** | **Cilium Or Flagellum Dependent Cell Motility** | 8.23268884255085e-18 | 1.53951281355701e-14 |
| **5** | **Microtubule Based Movement** | 2.02664117873762e-17 | 3.03185520339147e-14 |
| **6** | **Cilium Organization** | 5.51399984437944e-13 | 6.87411980599304e-10 |
| **7** | **Muscle Contraction** | 1.54439104784266e-12 | 1.44400562973289e-09 |
| **8** | **External Encapsulating Structure Organization** | 1.42513065961956e-12 | 1.44400562973289e-09 |
| **9** | **Microtubule Based Process** | 2.43202824878177e-12 | 2.02128570009863e-09 |
| **10** | **Muscle System Process** | 6.56877036983477e-12 | 4.91344023663641e-09 |
